# Evaluation of Sialyl-Lactotetra as a Marker for Epithelial Ovarian Tumors

**DOI:** 10.3389/fonc.2020.561888

**Published:** 2020-09-23

**Authors:** Angela Barone, Anna Linder, Constantina Mateoiu, Rasmus Köster Larsen, Ola Blixt, Susann Teneberg, Karin Sundfeldt

**Affiliations:** ^1^Department of Medical Biochemistry and Cell Biology, Institute of Biomedicine, Sahlgrenska Academy at University of Gothenburg, Gothenburg, Sweden; ^2^Department of Obstetrics and Gynecology, Sahlgrenska Cancer Centre, Institute of Clinical Sciences, Sahlgrenska Academy at University of Gothenburg, Gothenburg, Sweden; ^3^Department of Pathology and Cytology, Institute of Biomedicine, Sahlgrenska Academy at University of Gothenburg, Gothenburg, Sweden; ^4^Department of Chemistry, Faculty of Science, University of Copenhagen, Copenhagen, Denmark

**Keywords:** sialyl-lactotetra, glycosphingolipid characterization, biomarker, ovarian carcinoma, stem cell marker

## Abstract

Ovarian carcinoma is a heterogeneous disease with distinct molecular and histological profiles, ranging from low grade atypia to highly aggressive tumors associated with a poor prognosis. In the present study, glycosphingolipids were isolated from human high-grade serous ovarian carcinoma, whereby the novel stem cell marker Sialyl-lactotetra (S-Lc_4_) was characterized in two out of three cases. The presence and level of S-Lc_4_ was further evaluated immunohistochemically in a cohort of patients with ovarian tumors ranging from benign lesions to high grade serous carcinoma (*n* = 478). Its expression was assessed in association with tumor grade, stage, histology, and survival. The data showed that S-Lc_4_ is most common and highly expressed in borderline type tumors and carcinomas with low levels of aggressiveness, such as mucinous, endometrioid, and low grade serous. Accordingly, S-Lc_4_-positivity was associated with better disease-free survival. The expression of S-Lc_4_ was seemingly associated with lineage continuity and could be traced from premalignant lesions to carcinoma, suggesting inheritance by a stem cell lineage that gives rise to generally indolent tumors.

## Introduction

Ovarian carcinoma is the most lethal gynecological cancer (1). The high mortality is mainly associated with late discovery due to vague symptoms (2) and the high prevalence of recurrence (3). Ovarian carcinoma is a morphologically and molecularly heterogeneous disease (4). Despite the high diversity the same standard care is implemented consisting of aggressive surgery followed by chemotherapy. Recurrence is believed to be associated with the inability to eradicate the entire original tumor burden, and the presence of cancer cells in the residual tissue with stem-like properties that serve as ancestries of drug resistance and recurrent disease (5, 6).

It has been suggested that the cell surface markers used for definition and characterization of human pluripotent stem cells may also serve as markers for cancer detection or as targets of cancer therapy (7). Many such stem cell markers are based on cell surface carbohydrate epitopes, such as the widely used glycosphingolipids globopentaosylceramide/SSEA-3 and sialyl-globopentaosylceramide/SSEA-4, and also the glycoprotein TRA 1-60/TRA 1-81 markers (8).

The sialyl-lactotetra (S-Lc_4_; Neu5Acα3Galβ3GlcNAcβ) sequence was recently identified as a novel marker of undifferentiated human pluripotent stem cells, having a high cell surface expression on both human embryonic stem cells and human induced pluripotent stem cells, which is rapidly down-regulated upon differentiation (9, 10). In contrast, the distribution of sialyl-lactotetraosylceramide in normal human tissues is very limited. This ganglioside is only found in human meconium (11) and in the brains of young children, where it gradually disappears after 2 years of age (12). In addition, sialyl-lactotetraosylceramide has been found in some human cancers, such as small cell lung carcinoma (13), glioma (14, 15), and in embryonal carcinoma cells (16). More recently, sialyl-lactotetraosylceramide was also characterized in an ovarian cancer cell line (17). Additionally, S-Lc_4_ has recently shown promise as a marker for pancreatic carcinomas (18), suggesting clinical applications of S-Lc_4_ for epithelial tumors.

Ovarian tumors are classified based on their tissue of origin, where epithelial tumors are the predominant type. The epithelial tumors are further categorized based on atypia (benign, borderline, malignant) and histological appearance (low and high grade serous, mucinous, endometrioid, clear cell) (19). Sub-classification of serous carcinomas appertains to distinct molecular profile, histology, and behavior (20). High-grade serous carcinoma (HGSC) is the most common and lethal histology subtype and accounts for 65–70% of the diagnosed ovarian carcinomas. Less common are low-grade serous carcinomas (LGSC, <5%), endometrioid (10%), clear cell (10%), and mucinous carcinomas (3%) (2, 21, 22). An additional classification of ovarian carcinomas has been suggested. According to this dichotomous classification system, LGSC, endometrioid, clear cell, and mucinous carcinomas are designated Type 1, while HGSC are classified as Type 2 together with undifferentiated carcinomas and carcinosarcomas (20). Type 1 carcinomas, are suggested to be relatively indolent tumors that arise from precursor lesions such as endometriosis, benign, or borderline type tumors (20, 23–25).

Although Type 1 tumors frequently display low sensibility to chemotherapy, they are commonly diagnosed at early stages and consequently have a better prognosis. There is compelling evidence suggesting that Type 2 tumors, especially HGSC, stem from precursor lesions in the fallopian tube with the ovaries being the second site (26, 27). In comparison, these tumors progress more aggressively, and are initially chemosensitive but frequently acquire chemoresistance (28, 29).

In this study, we have evaluated the potential of S-Lc_4_ as a biomarker for ovarian tumors, by isolation and characterizing glycosphingolipids from three human HGSCs, and examination of the immunoreactivity of anti-S-Lc_4_ antibodies in a comprehensive tissue micro array of benign, borderline type and malignant ovarian tumor samples.

## Results

### Chemical Characterization of S-Lc_4_ in Ovarian Carcinomas

**Figure 1:**
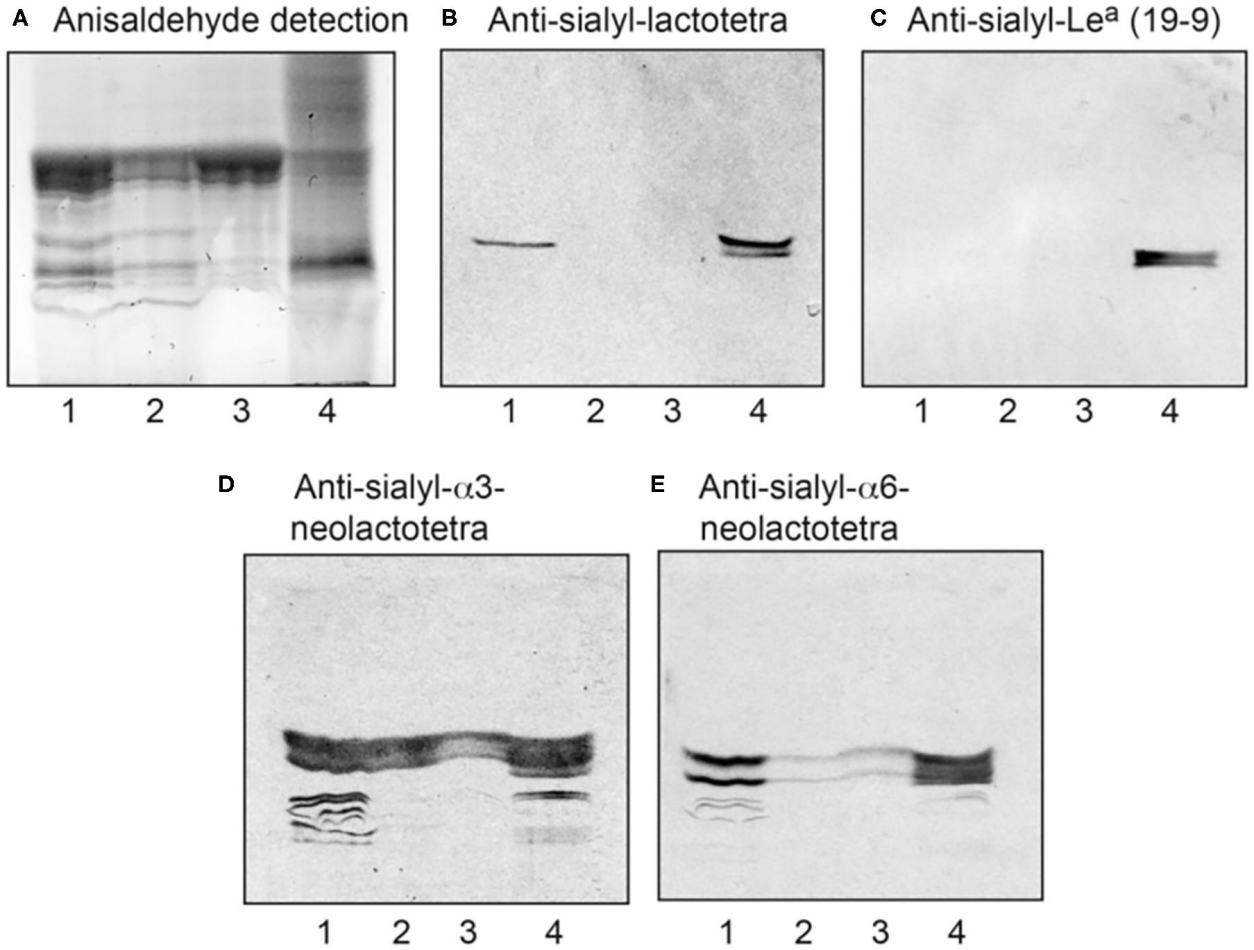
Characterization of the acid glycosphingolipids of ovarian cancers by binding of monoclonal antibodies. **(A)** Thin-layer chromatogram after detection with anisaldehyde, and autoradiograms obtained by binding of the monoclonal antibodies directed against **(B)** sialyl-lactotetra, **(C)** sialyl-Le^a^, **(D)** sialyl-α3-neolactotetra, and **(E)** sialyl-α6-neolactotetra. Lane 1, total acid glycosphingolipids of ovarian cancer (Case 1), 40 μg; Lane 2, total acid glycosphingolipids of ovarian cancer (Case 2), 40 μg; Lane 3, total acid glycosphingolipids of ovarian cancer (Case 3), 40 μg: Lane 4, reference total acid glycosphingolipids of liver cancer lung metastasis, 40 μg.

To determine whether SLc_4_ is expressed in ovarian carcinomas, acid and non-acid glycosphingolipids were isolated from three cases of HGSC by standard methods. The amounts obtained are given in Table 1. Thin-layer chromatography showed that the major compound of the three acid fractions migrated at the level of the GM3 ganglioside, and several more slow-migrating compounds were also seen (Figure 1A, lanes 1-3).

**Table 1:** Glycosphingolipid preparations.

**Case No**.	**Wet weight**	**Dry weight**	**Acid GSLs**	**mg acid GSLs/g dry weight**	**Non-acid GSLs**	**mg non-acid GSLs/g dry weight**
1. HGSC Stage 3C	94.9 g	15.5 g	17.1 mg	1.1	67.7 mg	4.4
2. HGSC Stage 2B	73.4 g	8.1 g	8.0 mg	0.9	34.0 mg	4.2
3. HGSC Stage 4B	228.8 g	33.5 g	32.4 mg	0.9	87.4 mg	2.6

*Table of the samples included for assessment of glycosphingolipid in fresh frozen HGSC tumor tissue. Abbreviations used in the table: GSLs, glycosphingolipids. HGSC, High grade serous carcinoma*.

The acid fractions from the three HGSC were first characterized by binding of a number of monoclonal antibodies (Supplementary Table 1). A distinct binding of the anti-S-Lc_4_ antibody, which binds to glycoconjugates with terminal Neu5Acα3Galβ3GlcNAc sequence, to the acid glycosphingolipids of Case 1 was observed (Figure 1B, lane 1, while no binding of the anti-sialyl-Le^a^ antibody, recognizing the related sequence Neu5Acα3Galβ3(Fucα4)GlcNAc, to the ovarian carcinoma glycosphingolipids occurred (Figure 1C, lanes 1-3).

In two out of three binding assays the anti-S-Lc_4_ antibody also bound to the acid glycosphingolipids of Case 2, whereas no binding to the acid glycosphingolipids of Case 3 occurred (not shown). There was also binding of the monoclonal antibodies directed against the sialyl-α3-neolactotetra (Neu5Acα3Galβ4GlcNAc) and sialyl-α6-neolactotetra (Neu5Acα6Galβ4GlcNAc) epitopes (Supplementary Table 1) to the acid glycosphingolipid fractions (Figures 1D,E, lanes 1-3), suggesting the presence of sialyl-α3- and sialyl-α6-neolactotetraosylceramides in all three cases.

**Figure 2:**
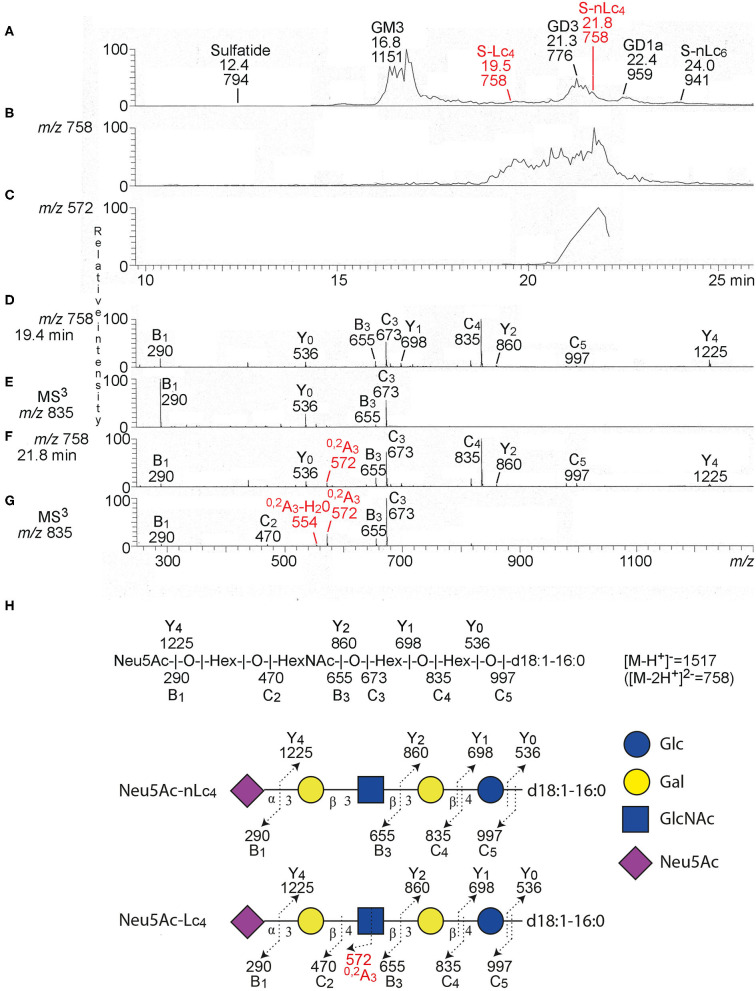
LC-ESI/MS of the total acid glycosphingolipids of ovarian cancer. **(A)** Base peak chromatogram from LC-ESI/MS of acid glycosphingolipids of ovarian cancer (Case 1). **(B)** Extracted ion chromatogram for *m/z* 757.5–758.5. **(C)** Extracted ion chromatogram for *m/z* 571.5–572.5. **(D)** MS^2^ of the ion at *m/z* 758 (retention time 19.4 min). **(E)** MS^3^ of the ion at *m/z* 835. **(F)** MS^2^ of the ion at *m/z* 758 (retention time 21.8 min). **(G)** MS^3^ of the ion at *m/z* 835. **(H)** Interpretation formulas. The proposed structures are depicted using the Symbol Nomenclature for Glycomics (SNFG) (30, 31), and nomenclature of fragments defined by Domon and Costello (32).The identification of glycosphingolipids was based on their retention times, determined molecular masses and subsequent MS^2^. Sulfatide, SO_3_-3Glcβ1Cer; GM3, Neu5Acα3Galβ4Glcβ1Cer; S-Lc_4_, Neu5Acα3Galβ3GlcNAcβ3Galβ4Glcβ1Cer; GD3, Neu5Acα8Neu5Acα3Galβ4Glcβ1Cer; S-nLc_4_, Neu5Acα3Galβ4GlcNAcβ3Galβ4Glcβ1Cer; GD1a, Neu5Acα3Galβ3GalNAcβ4(Neu5Acα3)Galβ4Glcβ1Cer; S-Lc_6_, Neu5Acα3Galβ4GlcNAcβ3Galβ4GlcNAcβ3Galβ4Glcβ1Cer.

Thereafter the total acid glycosphingolipid fractions of the three ovarian carcinomas were characterized by liquid chromatography electrospray ionization mass spectrometry (LC-ESI/MS). The base peak chromatograms obtained from the total acid glycosphingolipid fractions of one ovarian carcinoma (Case 1) (Figure 2A) had a number of singly charged ([M-H^+^]^−^) and doubly charged ([M-2H^+^]^2−^ ions) molecular ions, providing information about the molecular masses of the glycosphingolipids. The dominating ions were found at *m/z* 1151 and *m/z* 776, and minor molecular ions at *m/z* 794, *m/z* 758, *m/z* 959, and *m/z* 941 were also present (Supplementary Table 2). The identity of the glycosphingolipids was obtained by MS/MS (MS^2^), from which the carbohydrate sequence and ceramide composition was deduced. Thereby sulfatide with d18:1–h16:0 ceramide (*m/z* 794), the GM3 ganglioside with d18:1–16:0 ceramide (*m/z* 1151), the GD3 ganglioside with d18:1–24:1 ceramide (*m/z* 776), the GD1a ganglioside with d18:1–24:1 ceramide (*m/z* 959), and the ganglioside sialyl-neolactohexaosylceramide with d18:1–16:0 ceramide (*m/z* 941), were identified. A molecular ion at *m/z* 758 corresponds to a ganglioside with one Neu5Ac, one HexNAc, three Hex, and d18:1–16:0 ceramide. Searching for *m/z* 758 gave two ions eluting at 18.9–20.8 and 20.8–22.0 min, respectively (Figure 2B).

MS^2^ and MS^3^ of the late-eluting molecular ion at *m/z* 758 (retention time 21.8 min) identified a ganglioside with Neu5Ac-Hex-HexNAc-Hex-Hex carbohydrate sequence and d18:1–16:0 ceramide (Figures 2F,G). This was deduced from B and C ion series (B_1_ at *m/z* 290, C_2_ at *m/z* 470, B_3_ at *m/z* 655, C_3_ at *m/z* 673, C_4_ at *m/z* 835, and C_5_ at *m/z* 997), and the Y ion series (Y_0_ at *m/z* 536, Y_1_ at *m/z* 698, Y_2_ at *m/z* 860, and Y_4_ at *m/z* 1225) (Supplementary Table 3). The MS^2^ and MS^3^ spectra also had a ^0, 2^A_3_ ion at *m/z* 572, and a ^0, 2^A_3_-H_2_O ion at *m/z* 544 (Figures 2B,C,F,G). Cross-ring ^0, 2^A-type fragment ions are characteristic for 4-substituted HexNAcs, i.e., a type 2 carbohydrate chain (Galβ4GlcNAc) (33, 34). The spectral features thus allowed identification of sialyl-neolactotetraosylceramide (S-nLc_4_; Neu5Acα3Galβ4GlcNAcβ3Galβ4Glcβ1Cer).

A ganglioside with Neu5Ac-Hex-HexNAc-Hex-Hex carbohydrate sequence and d18:1–16:0 ceramide was also identified by MS^2^ and MS^3^ of the molecular ion at *m/z* 758 eluting at retention time 19.4 min (Figures 2D,E), by the series of B and C ions (B_1_ at *m/z* 290, B_3_ at *m/z* 655, C_3_ at *m/z* 673, C_4_ at *m/z* 835, and C_5_ at *m/z* 997), and Y ions (Y_0_ at *m/z* 536, Y_1_ at *m/z* 698, Y_2_ at *m/z* 860, and Y_4_ at *m/z* 1225) (Supplementary Table 2). This spectrum had no ^0, 2^A_3_ ion at *m/z* 572, which indicated a 3-substituted HexNAc, i.e., a type 1 carbohydrate chain (Galβ3GlcNAc). Thus, sialyl-lactotetraosylceramide (S-Lc_4_; Neu5Acα3Galβ3GlcNAcβ3Galβ4Glcβ1Cer) was identified (Figure 2H).

In the same manner sialyl-neolactotetraosylceramide and sialyl-lactotetraosylceramide with d18:1-24:1 ceramide were characterized by LC-ESI/MS of the acid glycosphingolipids isolated from Case 2 (Supplementary Figure 1), whereas Case 3 had only sialyl-neolactotetraosylceramide with d18:1-24:1 ceramide according to LC-ESI/MS (Supplementary Figure 2).

Thus, S-Lc_4_, which is not found in normal adult human tissues, was characterized in two out of three cases of HGSC by antibody binding and mass spectrometry. No glycosphingolipids with terminal Neu5Acα3Galβ3(Fucα4)GlcNAc sequence, recognized by the 19:9/anti-sialyl-Le^a^ antibodies, were characterized in the HGSCs. These findings prompted us to evaluate the distribution of S-Lc_4_ in an extended cohort of tumor samples including benign, borderline type, and malignant ovarian tissue biopsies.

### Localization and Distribution of S-Lc_4_ in Benign, Borderline Type, and Malignant Ovarian Tumors

**Figure 3:**
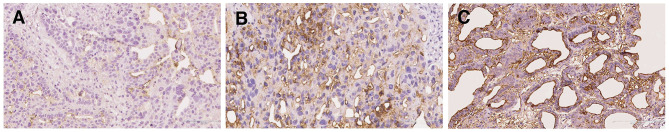
Immunohistochemical staining of S-Lc_4_ demonstrating differences in intensity. **(A)** Representative image of weak S-Lc_4_ immunohistochemical staining represented by High grade serous carcinoma. Scale bar 100 μm. **(B)** Representative image of moderate immunohistochemical staining represented by Endometrioid adenocarcinoma. Scale bar 100 μm. **(C)** Representative image of strong immunohistochemical staining represented by Low grade serous carcinoma. Scale bar 200 μm.

**Figure 4:**
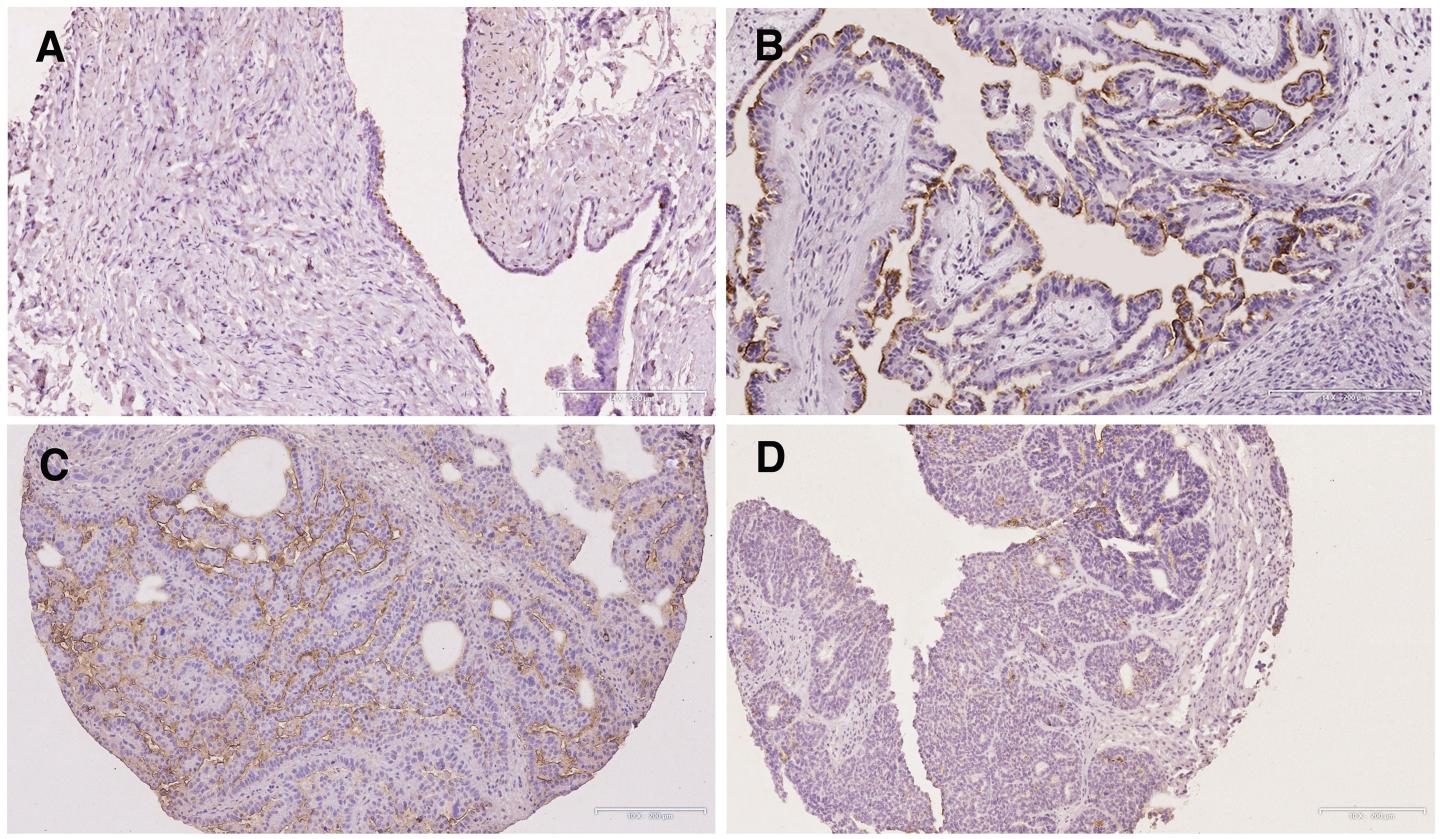
Immunohistochemical staining of S-Lc_4_ in ovarian tumors with different grade of malignancy. Representative images of immunohistochemical staining of carcinomas with different levels of malignancy. Tumor types include **(A)** Serous cystadenoma, **(B)** Borderline type serous tumor, **(C)** Low grade serous carcinoma, and **(D)** High grade serous carcinoma. Scale bar represent 200 μm.

**Figure 5:**
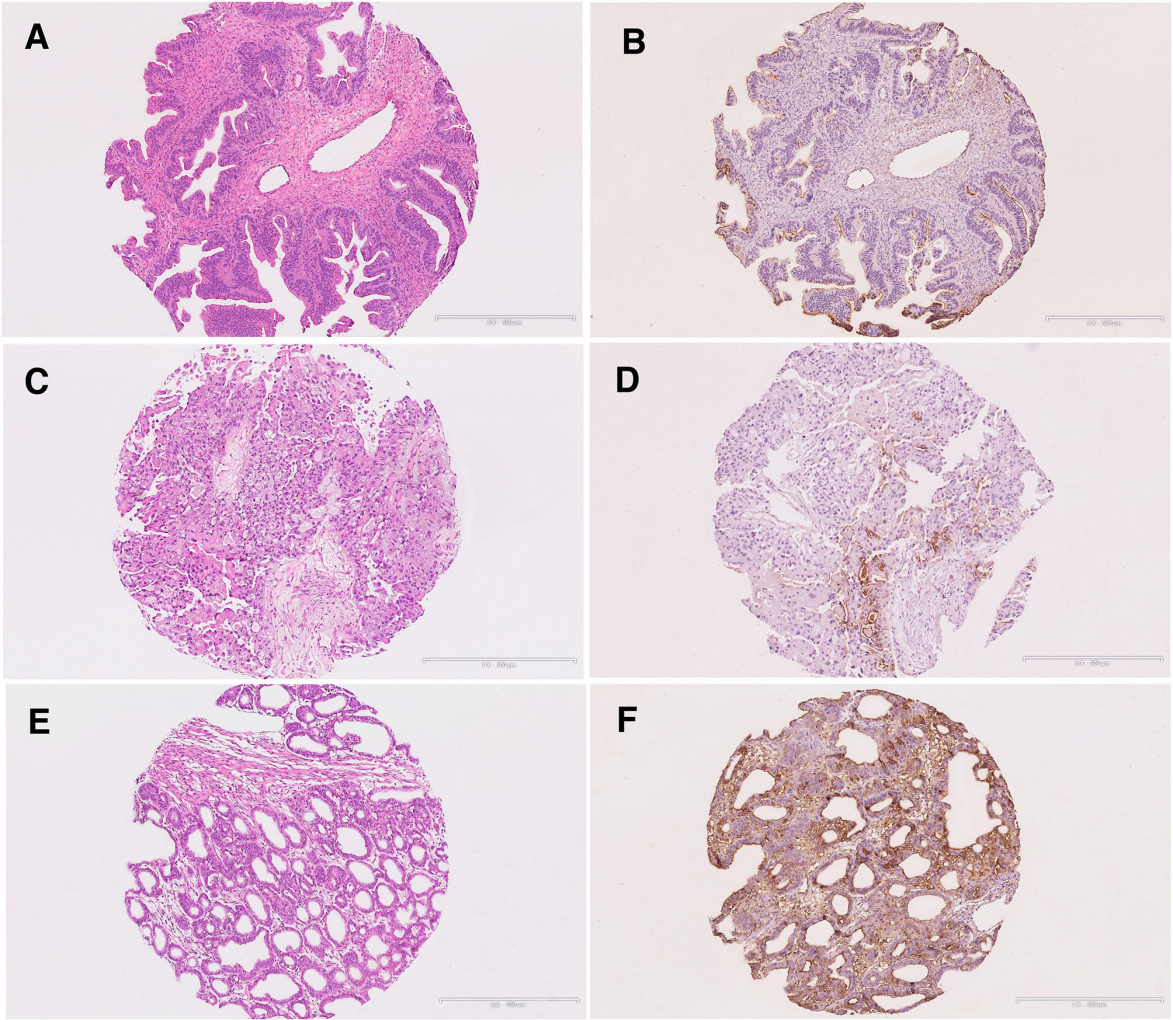
Immunohistochemical staining of S-Lc_4_ in Type 1 ovarian carcinoma. **(A,B)** Hematoxylin-eosin and S-Lc_4_ immunohistochemical staining in Mucinous carcinoma. **(C,D)** Hematoxylin-eosin and S-Lc_4_ immunohistochemical staining in Clear cell carcinoma. **(E,F)** Hematoxylin-eosin and S-Lc_4_ immunohistochemical staining in Endometrioid carcinoma. Scale bar represent 500 μm.

To evaluate the distribution, intensity, and localization of S-Lc_4_ in ovarian tumors, the staining of the S-Lc_4_ antigen was assessed with immunohistochemistry in a cohort of consecutively collected ovarian tumors operated for suspicious pelvic mass or ovarian cyst, previously described (35). S-Lc_4_ antibody showed affinity for the apical surface of ovarian tumors epithelial cells and limited staining of the ovarian tumor stroma, which was considered unspecific. Intratumoral heterogeneity in immunohistochemical staining was scored based on the percentage of tumor cells stained (score 0–3) and staining intensity (none, weak, moderate, or strong) (Figure 3). We noticed significant intensity variation between benign, borderline type, and malignant tumors, visualized by serous tumors of different grades of malignancy (Figure 4). Other malignant epithelial ovarian tumors classified in the dualistic model as Type 1 tumors, that is mucinous, clear cell, and endometrioid carcinomas, also displayed the S-Lc_4_ antigen in a specific apical surface pattern (Figure 5). Overall, the results showed that apparent variations in the expression of S-Lc_4_ were associated with different grades of malignancy and histologic subtype.

### Frequency, Distribution, and Statistical Evaluation of S-Lc_4_ in Ovarian Tumors

The immunohistochemical evaluation discovered anti-S-Lc_4_ positive cells in 216 cases (44.4%) and the mean H-score value and SEM was 0.896 + 0.064 (range: 0–5.7). The frequency of S-Lc_4_ varied significantly between tumor atypia, and histology (Table 2). There was no correlation between age at diagnosis and S-Lc_4_ expression in the overall data set (Spearman's rho −0.066, 0.206), or in the malignant group. (Spearmans's rho −0.034, *p* = 0.589). There was a high frequency of S-Lc_4_ in borderline type and malignant tumors, compared to benign tumors and metastases (Table 2). For borderline type tumors, the serous histology displayed a higher presence of S-Lc_4_ compared to mucinous. Only two borderline type tumors of endometrioid histology were available, this group was thus omitted from further analysis (Table 2; *p*-value for analysis including endometrioid histology in parenthesis). For the malignant group, HGSC displayed a comparatively low frequency of S-Lc_4_ compared to other histologies. In line, the presence of S-Lc_4_ in Type 2 was significantly (*p* < 0.001) lower than that of Type 1 tumors.

**Table 2:** Frequency of S-Lc_4_ in association with clinicopathological characteristics.

**Characteristic**	**No. Pts**	**No.Cases with pos S-Lc_**4**_**	*p*-value^*^
	**(%)**	**(%)**	
**Malignancy**			< 0.0001^*^
Benign	137	38	
	(27.9)	(27.7)	
Borderline	75	44	
	(15.9)	(58.7)	
Malignant	239	118	
	(50.5)	(49.4)	
Metastases	27	9	
	(5.7)	(33.3)	
**Histology (benign)**			0.113
Endometriosis	4	3	
	(3.2)	(75.0)	
Mucinous	45	12	
	(35.7)	(26.7)	
Serous	77	21	
	(61.1)	(27.3)	
**Histology (borderline)**			0.016^*^ (0.025^*^)
Endometrioid	2	2	
	(2.7)	(100)	
Mucinous	32	14	
	(43.8)	(8, 36)	
Serous	39	28	
	(53.4)	(71.8)	
**Histology (malignant)**			0.015^*^
Endometrioid	21	14	
	(8.8)	(66.7)	
Mucinous	19	12	
	(7.9)	(63.2)	
LGSC	22	14	
	(9.2)	(63.6)	
HGSC	163	68	
	(68.2)	(41.7)	
Clear cell carcinoma	14	10	
	(5.9)	(71.4)	
**Stage (malignant)**			0.085
I	65	37	
	(27.2)	(56.9)	
II	18	11	
	(7.5)	(61.1)	
III	137	58	
	(57.3)	(42.3)	
IV	19	12	
	(7.9)	(63.2)	
**Type**			< 0.0001^*^
Type 1	75	50	
	(31.4)	(66.7)	
Type 2	164	68	
	(68.6)	(41.5)	
**Malignant (serous)**			0.079
LGSC	20	13	
	(10.9)	(65.0)	
HGSC	164	68	
	(89.1)	(41.5)	

*The number and percent (%) of patients with different characteristics as stated, listed together with the frequency of S-Lc_4_ positive specimens for that characteristic. The correlation between the presence of S-Lc_4_ and the clinicopathological characteristics was evaluated using Fishers' exact test or Person's Chi-square test, where applicable*.

* *Denotes statistically significant p-values*.

**Figure 6:**
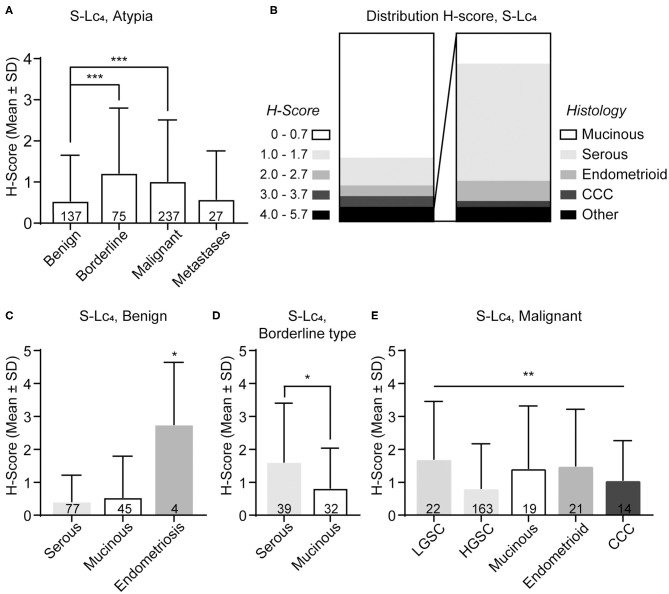
Distribution of S-Lc_4_ in epithelial ovarian tumors of different histologic subtypes. **(A)** Assessment of the S-Lc_4_ expression at different levels of malignancy. **(B)** Distribution of the level of S-Lc_4_ expression in the full data set (*n* = 478). Insertion demonstrate the histology distribution in tumors with high levels of S-Lc_4_ (*n* = 34). The level of S-Lc_4_ expression in different histology types compared per level of malignancy **(C)** Benign tumors, **(D)** Borderline type tumors, and **(E)** Malignant tumors. Data are presented as mean ± SD. *P*-values was determined with Mann-Whitney *U*-test or Kruskal-Wallis test, where applicable. Multiple comparisons was adjusted for by using Bonferroni correction. Significant *p*-values are denoted as; *p < 0.05, **p < 0.001, ***p < 0.0001. *P*-values represent pairwise significant data **(A,C)** or the Kruskal-Wallis test, when pairwise comparisons were not significant **(E)**. Number of patients are indicated in the figure.

The majority of the specimens displayed either no or small amounts of S-Lc_4_. Only 7% (34 out of 478) displayed a high level of S-Lc_4_ (H-score of ≥4), of these the majority had a serous histology (Figure 6A). S-Lc_4_ had a significantly higher expression in borderline type and malignant tumors compared to benign tumors (Figure 6B; Kruskal-Wallis test, adjusted *p* = 0.001 and < 0.0001, respectively). There was no significant difference between expression of S-Lc_4_ when comparing tumors of borderline type and malignant tumors. There was no evident variance when comparing the expression of S-Lc_4_ for different histological types in the overall data. However, further assessment of S-Lc_4_ in association with clinicopathological characteristics showed that there was a significant higher expression of S-Lc_4_ in endometriosis compared to the benign adenomas (mucinous and serous) (Figure 6C; *p* = 0.023). In the borderline type tumors, there was a significantly higher expression of S-Lc_4_ in serous tumors compared to those with a mucinous histology (Figure 6D; *p* = 0.024).

**Figure 7:**
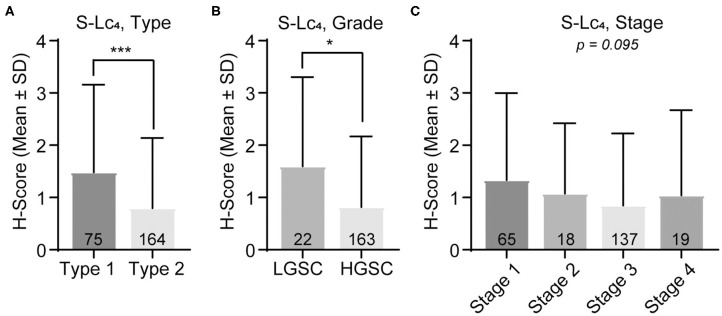
Assessment of S-Lc_4_ expression in ovarian carcinoma Type 1-2 and stage I-IV. **(A)** Assessment of the S-Lc_4_ expression in **(A)** Type 1 and Type 2 tumors. **(B)** Evaluation of the S-Lc_4_ expression in serous ovarian carcinoma, comparing LGSC and HGSC. **(C)** Evaluation of SL-c_4_ expression in different tumor stages. Data is presented as mean ± SD. Number of patients per group are indicated in the figure. Significant *p*-values are indicated as *p < 0.05, ***p < 0.0001. *P*-values were determined with Mann-Whitney test or Kruskall-Wallis with Bonferroni correction, where applicable. Number of patients in the groups are indicated in the figure.

Within the malignant group, the results showed that there was a significant difference in S-Lc_4_ expression between the histology subtypes (Figure 6E; *p* < 0.0094), however pairwise comparisons were not significant after correction for multiple comparisons. In the dualistic model, there was a significantly higher expression of S-Lc_4_ in Type 1 tumors compared to the more aggressive Type 2 tumors (Figure 7A, *p* < 0.0001) as well as higher expression in LGSC compared to HGSC (Figure 7B, *p* = 0.014). Further assessment of S-Lc_4_ expression in ovarian carcinoma suggested that stage III tumors displayed a generally lower expression compared to stage I, II, and IV, however this was not statistically significant (Figure 7C, *p* = 0.095).

### Evaluation of S-Lc_4_ as a Prognostic Marker for Ovarian Carcinoma

**Figure 8:**
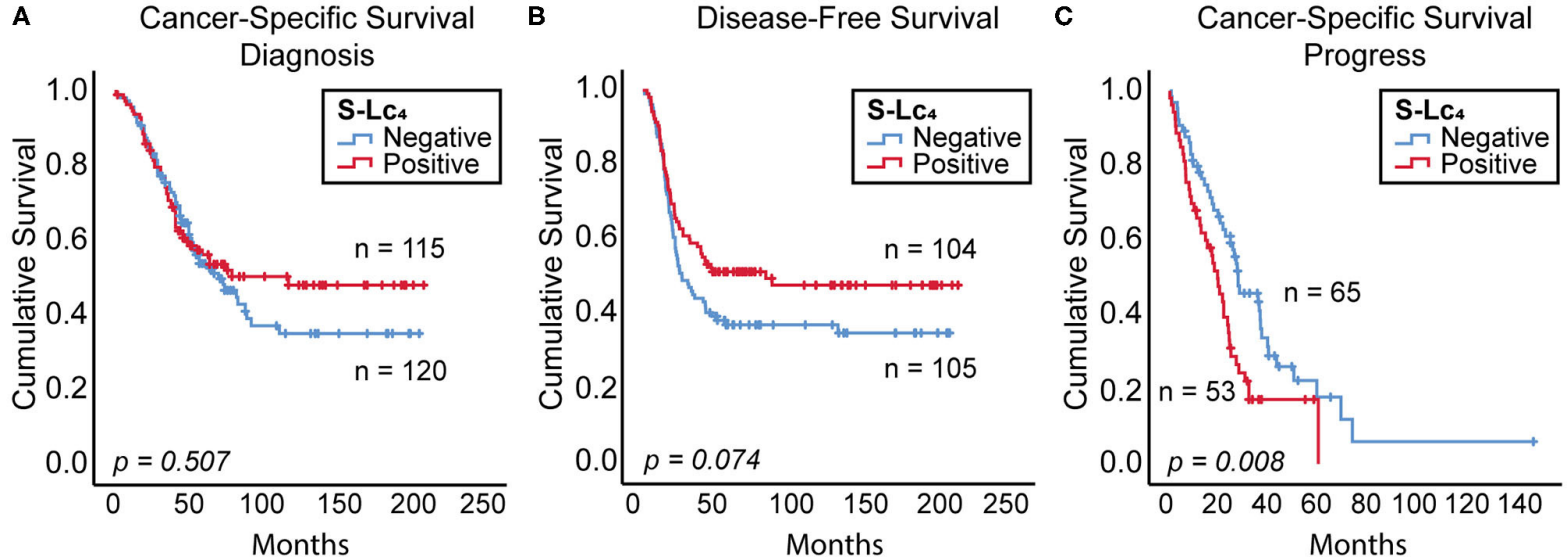
Prognostic value of S-Lc_4_ in ovarian carcinoma. Kaplan-Meier curve demonstrating the association between S-Lc_4_ expression and prognosis in terms of **(A)** cancer-specific survival (from time of diagnosis), *n* = 235, **(B)** Disease-free survival, *n* = 209, and **(C)** Cancer-specific survival (from time of progress), *n* = 118. The *p*-values indicated in the figure are determined using the log-rank test. Number of patients per group are indicated in the figure.

The prognostic impact of the expression of S-Lc_4_ was assessed in association with cancer-specific survival (CSS) and disease-free survival (DFS) from diagnosis. The Kaplan-Meier curve with log rank (*p* = 0.507) showed that there was no significant correlation between S-Lc_4_ expression and CSS, when comparing S-Lc_4_ negative and positive tumors (Figure 8A). However, considering DFS, the data suggests that S-Lc_4_ positive tumors progress more slowly (Figure 8B). The mean DFS time was 87.6 (95% CI: 70.294–107.999) months for patients with S-Lc_4_ negative tumors compared to 113.731 (95% CI: 95.133–132.491) for patients with S-Lc_4_ positive tumors. In the S-Lc_4_ negative group, the 5-year DFS was 37% (SE 4.8%) compared to 51% (SE 4.9%) in the positive group. Cox regression analysis confirmed a favorable prognostic value. For the continuous variable, the hazard ratio (HR) was 0.858 (95% CI: 0.747–0.985), *p* = 0.030. However, the significantly different expression in Type 1 and Type 2, and its potential prognostic implication, motivated stratification for this variable. The data showed that the prognostic value did not persist after stratification by tumor Type (HR 0.908, *p* = 0.180).

In contrast to the favorable prognosis considering DFS, the cancer-specific mortality considered from the time of progress was significantly reduced for patients with S-Lc_4_ expressing tumors (Figure 8C). The mean CSS was 37.7 months (95% CI: 26.295–49.136) in the S-Lc_4_ negative while 22.8 months (95% CI: 17.445–28.236) in the positive group. The 2-year survival after progress in the S-Lc_4_ positive group was 33.1% (SE 6.7%), compared to 59.3% (SE 6.3%) in the S-Lc_4_ negative group. Cox regression analysis for the continuous variable showed that the HR was 1.184, *p* = 0.036 (95% CI: 1.011–1.386). The prognostic value remained significant after stratification for tumor Type. HR after stratification was 1.190, *p* = 0.033 (95% CI: 1.014–1.396).

### Evaluation of Serum S-Lc_4_ Antibodies in Women With Ovarian Tumors

We performed glycan microarray analysis to investigate the presence of serum IgG and IgM antibodies targeting S-Lc_4_. Grouping of patients were conducted based on histology and S-Lc_4_ positivity (see material and methods). We did not observe any significant difference in autoantibody responses comparing the four oligosaccharides S-Lc_4_, sialyl-α3-neolactotetra, sialyl-Le^a^ and sialyl-Le^x^ (Supplementary Figure 3) between the analyzed groups.

## Discussion

To the best of our knowledge, this is the first time S-Lc_4_ expression has been evaluated in ovarian tumors and the first time glycosphingolipids have been isolated from human high grade serous ovarian carcinoma. The acid glycosphingolipids were characterized by antibody binding and mass spectrometry. Thereby, sialyl-lactotetraosylceramide was characterized in two of the three HGSC. Thereafter the immunoreactivity of anti-S-Lc_4_ was evaluated in a large cohort of patients with ovarian benign, borderline, and malignant tumors.

Recently the sialyl-lactotetra (S-Lc_4_) carbohydrate sequence (Neu5Acα3Galβ3GlcNAc) was identified as a novel marker of human pluripotent stem cells (9, 10). S-Lc_4_ is specifically expressed by undifferentiated human pluripotent stem cells, both embryonic stem cells and induced pluripotent stem cells, and its expression decreases upon early differentiation. In the present study, we assessed the potential value of S-Lc_4_ as a marker for ovarian carcinomas. The data showed that accumulation of S-Lc_4_ expressing cells was not a general age-related event, but tumor specific, suggested by the lack of correlation between age and S-Lc_4_ expression in both the total cohort and the malignant group. Furthermore, the data showed that a small fraction of S-Lc_4_ expressing cells were present in ovarian tumors of all levels of malignancy. However, S-Lc_4_ expression was less frequent in benign tumors. Interestingly, the data showed that the stem cell marker S-Lc_4_ was most frequently expressed in comparatively indolent tumors with low cancerous potential (19, 37), such as serous borderline type and Type 1 tumors.

Additionally, the results propose that endometriomas express high levels of S-Lc_4_, suggesting that the marker can be traced to a non-malignant precursor lesion. Despite the low number of endometriosis cases in the present study, this potential finding is interesting considering the shortage of markers for this disease (38). However, the significance of this observation needs to be confirmed and prospective studies are warranted. Considering the ancestry of Type 1 tumors, alleged to descend from endometriosis and borderline tumors (23–25), these data may suggest that the S-Lc_4_ expression is preserved in tumors that originate from stem cell lineages that give rise to low proliferative tumors.

There are only a few previous studies of glycosphingolipids of human ovarian carcinomas (39). Kiguchi et al. reported that the gangliosides GM3 (Neu5Acα3Galβ4Glcβ1Cer) and GD3 (Neu5Acα8Neu5Acα3Galβ4Glcβ1Cer) are major gangliosides of both the normal human ovary and ovarian tumors. The gangliosides were here identified by co-migration with reference gangliosides on thin-layer plates. Other findings were high levels of sulfatide (SO_3_-3Galβ1Cer), and non-acid glycosphingolipids with terminal Le^a^ (Galβ3(Fucα4)GlcNAc-) and Le^b^ (Fucα2Galβ3(Fucα4)GlcNAc-) determinants in mucinous cystadenocarcinomas. In addition, the P1 glycosphingolipid (Galα4Galβ4GlcNAcβ3Galβ4Glcβ1Cer) has been identified by LC-ESI/MS of glycosphingolipids from serous ovarian cancers (40). The presence of tumor-derived S-Lc_4_ in plasma of patients with pancreatic cancers was recently shown, demonstrating that this glycan is a novel liquid biopsy biomarker for some subsets of pancreatic cancers, as accurate as the cancer antigen CA19-9 (41). The present data however suggests that this may not the case for ovarian carcinoma, as no significant difference in immunoreactivity was detected in patient serum, irrespective of histology or S-Lc_4_ tissue positivity.

It has been shown that spatially and morphologically distinct subsets of pancreatic cancer cells expressed S-Lc_4_ (here denoted sTRA) or the CA19-9 antigen (18, 42). Well-differentiated ductal pancreatic adenocarcinomas typically expressed both glycans, whereas just one of the markers was expressed by poorly differentiated tumors. Patients with higher dual staining of CA19-9 and S-Lc_4_/sTRA had statistically longer time-to-progression after surgery. In agreement with the association between S-Lc_4_ and a less aggressive type of pancreas cancer, the present data suggests that S-Lc_4_ was a moderate favorable predictive marker in terms of progress-free survival. However, considering the excess of HGSC, that account for almost 70% of the tumors in the present data set, this result is probably reflective of the generally indolent course of Type 1 tumors, compared to the exceptionally aggressive HGSC (29, 37). In agreement, the prognostic value of S-Lc_4_ was no longer significant after stratification for tumor Type.

Although cancer stem cells are primarily considered within the context of a negative prognosis, the expression of stem-cell markers and favorable outcomes has previously been described for various types of cancer (43–45). This includes ovarian carcinoma where higher levels of recognized ovarian cancer stem cell markers such as CD44 (46) and ALDH1 (36) were shown in association with extended progression-free and overall survival. This was described in association with high levels in well-differentiated tumors compared to tumors of low differentiation, consistent with the data presented here. A key factor behind the high mortality of ovarian cancer is the high prevalence of treatment-resistant tumor growth (3). Thus, the occurrence of stem cells in ovarian carcinoma has gained high interest considering the pivotal role in association with tumor recurrence and aggressive progression (5, 6). The data presented here suggests that S-Lc_4_ is associated with a good treatment response and prolonged time to regression in a histologic tumor subtype related fashion. However, the results also suggest that, independently of histology or stage, the survival time after progress was significantly shorter for patients with S-Lc_4_ expressing tumors compared to S-Lc_4_ negative. Taken together, this data proposes that S-Lc_4_ was indicative of a subset of ovarian cancer that acquires an exceedingly aggressive phenotype after relapse.

In conclusion, S-Lc_4_ was expressed by all ovarian tumor types evaluated. However, Sialyl-lactotetra was both more frequent and expressed at a higher level in borderline type and malignant ovarian tumors. The exception was HGSC, which generally displayed a significantly lower expression. Importantly, the expression of S-Lc_4_ does not increase over time, but its level and frequency is cancer-related. The current data suggests that S-Lc_4_ could be of value as a marker for serous borderline type and Type 1 tumors. The lack of molecular markers for these tumor types suggests that these findings could be of clinical value. However, prospective studies are required to evaluate these findings.

## Materials and Methods

### Patient and Tumor Characteristics

#### Patients Included for Assessment of Glycosphingolipids

Fresh tumor tissue, 5–8 cm diameter, were obtained from three patients that underwent primary debulking surgery at the Sahlgrenska University hospital, due to a large ovarian mass with high suspicion of ovarian cancer (11–2016, 12–2016, and 11–2017) (Table 1). Microscopic evaluation was performed by subspecialist in gynecologic pathology (CM), and high grade serous carcinoma (HGSC) was confirmed in each case (*See* Table 1). Case 1; HGSC, stage IIB, case 2; HGSC, stage IVB, case 3; HGSC, stage IIIC.

#### Study Cohort for Tissue Micro Arrays and Immunohistochemical Analysis

Four hundred and seventy-eight patients were included in the present study, of these 137 patients had benign diseases of the ovary, 75 had borderline type, 239 had malignant tumors, and 27 had ovary-localized metastases. For comprehensive composition of the TMA see Table 2. The mean age for the overall cohort was 59.6 years (Range: 16–88). The mean age in the patient groups of ovarian tumors was benign 64.3 years (Range: 28–88), borderline type 50.6 years (Range: 16–85), and malignant 61.6 years (Range: 28–88). Median follow up time of 49.5 months (Range: 1–208), was considered from time of diagnosis until death or 22-may-2019.

In the group of patients with ovarian cancer, 49.2% (116 out of 236) women died from their disease, 41.5% (98 out of 236) were still alive at the time of the last evaluation and 9.3% (22 out of 236) patients died from unknown or unrelated causes. The survival time was defined from date of primary surgery to date of death. The progress status was known for 272 patients, and 126 of these progressed during this survey. National treatment guidelines with protocols for standard surgery procedures (staging and adequate debulking cytoreductive surgery) were followed for all patients. Clinicopathological information for the cohort was obtained from the Cancer Registry at the National Board of Health and Welfare (Stockholm, Sweden) and the National Quality Registry at the Regional Cancer Centre West (Gothenburg, Sweden). Progress was defined by RECIST criteria (47). Disease-free survival (DFS), was defined as date of primary surgery to date of confirmed progress.

#### Patients Included for Glycan Array

Serum anti-S-Lc_4_ was evaluated in patients that in the tissue micro arrays showed positive S-Lc_4_ staining (*n* = 20) and negative S-Lc_4_ staining (*n* = 20) and included 10 cases with low- and high-grade serous adenocarcinoma and 10 cases with serous and mucinous borderline type tumors each. The corresponding serum was collected at the time of primary diagnosis in all patients. The controls comprised of serum from healthy controls, without known cancer, retrieved from the blood bank (*n* = 66 for IgG and 22 for IgM).

### Tissue Micro Array and Scoring

The antibody used is given in Supplementary Table 1. IHC staining for S-Lc_4_ was performed on 13 tissue microarrays (TMAs) containing ovarian tumor specimens from individual women. The TMAs were constructed from cases collected from the Sahlgrenska Gynecology tumor bank. The TMAs were constructed using 3 replicate 1 mm cylindrical core biopsies from each case. TMAs were designed by study authors (KS and CM) and constructed in house (BW). The whole section was digitally scanned with Leica SCN400 (Leica Microsystems, Milton Keynes, UK) and analyzed using SlidePath Gateway Client LAN software. Diagnostic confirmation was based on morphologic review (CM) of original hematoxylin and eosin slides and any accompanying IHC stains using criteria based on the 2014 World Health Organization Classification of Gynecologic Tumors.

Five micrometer sections of formalin-fixed paraffin-embedded (FFPE) tissue were tested for the presence of S-Lc_4_ using anti-sialyl-lactotetra (clone TR4). Scoring of S-Lc_4_ positivity was performed using the additive Quick semiquantitative method, which combines staining intensity and percentage of tumor cell staining. For intensity level a score from 0 to 3 was given (0 = none, 1 = weak, 2 = moderate, 3 = strong). The proportion of malignant cells staining positively throughout the section was assigned scores from 0 to 3 (0 <5% positive cells, 1 = 5–10% positive cells, 2 = 11–50% positive cells, 3 >51% positive cells). On the TMA sections percentage of positive tumor cells was appreciated based on positive staining in every tissue core individually. The scores are summed to give a maximum H-score of 6.

### Glycosphingolipid Preparations

The three HGSC were lyophilized, and acid and non-acid glycosphingolipids were thereafter isolated as described (48). The first step was Soxhlet extraction with chloroform and methanol (first 2:1 by volume for 24 h, and thereafter 1:9 by volume for 24 h). The extracts were pooled and the material was subjected to mild alkaline hydrolysis and dialysis. Thereafter the material was separated on a silicic acid column. This was followed by chromatography on a DEAE-cellulose column giving acid and non-acid glycosphingolipid fractions. Separation of the non-acid glycosphingolipids from alkali-stable phospholipids was done by acteylation of the material and chromatography on a second silicic acid column, followed by deacetylation and dialysis. Chromatographies on DEAE-cellulose and silicic acid columns were done for further purifications. The acid fractions from the two DEAE-cellulose columns were pooled, and further purified by chromatography on a silicic acid column eluted with increasing amounts of methanol in chloroform. Table 1 gives the amounts of acid and non-acid glycosphingolipids obtained from each tumor.

### Chromatogram Binding Assays

Isolation and characterization of reference glycosphingolipids was done as described (48). For thin-layer chromatography aluminium- or glass-backed silica gel 60 high performance thin-layer chromatography plates (Merck) were used. Glycosphingolipid mixtures (40 μg) were applied on the plates, and developed with chloroform/methanol/water (60:35:8, by volume). The anisaldehyde reagent was used for chemical detection (48). The mouse monoclonal antibodies used in the chromatogram binding assays are given in Supplementary Table 1. Antibody binding to the glycosphingolipids on thin-layer chromatograms was done as described Barone et al. (9). In short, aluminium-backed thin-layer plates with separated glycosphingolipids were dipped for 1 min in diethylether/*n*-hexane (1:5, by volume) containing 0.5% (w/v) polyisobutylmethacrylate (Sigma-Aldrich). The chromatograms were dried and then covered with phosphate-buffered saline, pH 7.3 (PBS), containing 2% bovine serum albumin and 0.1% NaN_3_ (Solution A), and left for 2 h at room temperature. Thereafter the chromatograms were incubated with monoclonal antibody suspensions (the dilution of each antibody are given in Table EV1) for 2 h at room temperature. The chromatograms were then washed with PBS, and thereafter followed another 2 h incubation with ^125^I-labeled rabbit anti-mouse antibodies (DakoCytomation Norden A/S, Glostrup, Denmark). These antibodies were labeled by the Iodogen method according to the manufacturer's (Pierce) instructions, and diluted to 2 × 10^6^ cpm/ml in Sol. A. The final step was washing the chromatograms with PBS, and after drying the chromatograms were autoradiographed for 12–24 h using XAR-5 x-ray films (Carestream; 8941114).

### LC-ESI/MS of Native Glycosphingolipids

The native non-acid glycosphingolipid fractions were analyzed by LC-ESI/MS as described (49). Data acquisition and processing were done using Xcalibur software (Version 2.0.7). Glycosphingolipid sequences were assigned manually on the basis of knowledge of glycosphingolipid biosynthetic pathways, with the assistance of the Glycoworkbench tool (Version 2.1), and by comparing with the retention times and MS^2^ spectra obtained of reference glycosphingolipids.

### Glycan Array

Printing of the microarray slides was performed using a BioRobotics MicroGrid II spotter (Genomics Solution) using Stealth 3BMicro Spotting Pins with a deposit volume of ~6 nL of glycopeptide in print buffer (150 mM phosphate, 0.005% CHAPS pH 8.5). The compounds were distributed (20 μL per well) in 384-well-source plates (BD Falcon MicrotestTM 384-well 30 μL assay plates from BD Biosciences, Le Pont De Claix, France) and printed in three replicates using an 8-pin (2°ø 4) configuration within a 15 × 15 subgrid at a 0.21 mm pitch between each spot. The pin dwell time in the wells was 4 s and the pins underwent three wash cycles in between source plate visits. The complete 2 array pattern was printed on a 16-well-slide in duplicate, distributed in two columns and eight rows. Immediately after printing, the slides were incubated at 80% humidity for 60 min. Remaining NHS groups on the slides were blocked by immersion in the blocking buffer (50 mM ethanolamine in 50 mM borate buffer, pH 9.2) for 1 h. Slides were rinsed in Millipore water, dried by centrifuging, and probed as described below. Slides were mounted into a 16-well FAST frame slide holder (Whatman). Diluted serum (100 μL of 1/30) in PBS-T (0.5 M NaCl, 3 mM KCl, 1.5 mM KH2PO4, 6.5 mM Na2HPO4, 1% BSA, 1% Triton-X-100, pH 7.4) was applied onto the slide and incubated overnight in an airtight container at 100% humidity. After primary incubation the slides were thoroughly washed in PBS-T (0.05% tween), extra care was taken to prevent dehydration of the slides. Secondary incubation was carried out as described above. Anti-IgM or anti-IgG was diluted in PLI-P at a 1/500 ratio. Scanning of the slides was performed on ScanArray, Microarray Scanner (Perkin Elmer) followed by image analysis with ScanArray Express 4.0 software (Perkin Elmer). Data was analyzed and plotted using R.

### Statistics

Statistical analysis was carried out using the IBM SPSS statistics, version 24. For all test ^*^p ≤ 0.05, ^**^p ≤ 0.01, ^***^p ≤ 0.001 were considered significant. The Mann-Whitney *U*-test or Kruskal-Wallis test was used for statistical evaluation of differences between one or more groups, respectively. Adjustment for multiple comparison was performed with the Bonferroni method. Categorical S-Lc_4_ expression in relation to clinicopathological characteristics was evaluated using Fisher's exact test or Chi-square test, where appropriate. Spearman rank correlation was used for evaluation of correlation between S-Lc_4_ expression and age. For CSS and DFS, Kaplan-Meier chart were created and present together with the Log-rank *p*-value and mean survival time 95% CI. The data was dichotomized in S-Lc_4_ negative or positive tumors (equivalent to the median score). Survival time was calculated from date of surgery or date of progress, as specified in the text. Univariable Cox regression was performed to calculate HR for the continuous S-Lc_4_ variable, data is presented with the 95% CI.

### Ethics Statement

The study was approved by the ethics committee of the Sahlgrenska University hospital (Dnr 201-15). Informed consent was obtained from all participants according to ethical guidelines.

### Additional Information

The glycosphingolipid nomenclature follows the recommendations by the IUPAC-IUB Commission on Biochemical Nomenclature (CBN for Lipids: *Eur. J. Biochem*. (1998) **257**, 293). It is assumed that Gal, Glc, GlcNAc, GalNAc, and Neu5Ac are of the D-configuration, Fuc of the L-configuration, and all sugars are present in the pyranose form.

In the shorthand nomenclature for fatty acids and bases, the number before the colon refers to the carbon chain length and the number after the colon gives the total number of double bonds in the molecule. Fatty acids with a 2-hydroxy group are denoted by the prefix h before the abbreviation e.g., h16:0. For long chain bases, d denotes dihydroxy and t trihydroxy. Thus, d18:1 designates sphingosine (1,3-dihydroxy-2-aminooctadecene) and t18:0 phytosphingosine (1,3,4-trihydroxy-2-aminooctadecane).

## Data Availability Statement

The original contributions presented in the study are publicly available. This data can be found here: https://glycopost.glycosmos.org/entry/GPST000076.

## Ethics Statement

This studies involving human participants were reviewed and approved by The Ethics Committee of Sahlgrenska University Hospital. The patients/participants provided their written informed consent to participate in this study.

## Author Contributions

ST, AB, and KS initiated the study. AB performed the tumor specific glycosphingolipid analysis. CM performed the microscopic evaluation and IHC scoring. AL analyzed the IHC data and performed the statistical analysis. RK and OB performed the serum S-Lc_4_ analysis. All authors took part in data evaluation, writing, and finalization of the manuscript.

## Conflict of Interest

The authors declare that the research was conducted in the absence of any commercial or financial relationships that could be construed as a potential conflict of interest.
