# Supplementary material for: Evaluation of Sialyl-Lactotetra as a Marker for Epithelial Ovarian Tumors
Source: Front Oncol. 2020 Sep 23;10:561888. doi: 10.3389/fonc.2020.561888 (PMC7539041; doi:10.3389/fonc.2020.561888)
Supplement: Supplementary file 1 [file Data_Sheet_1.PDF]

# Supplementary Data

**Table of content**


1. **Table S1** Monoclonal Antibodies
2. **Figure S1** Characterization of the acid glycosphingolipids (Case 2)
3. **Table S2** Theoretical masses and *m/z* of glycosphingolipids (Case 1)
4. **Table S3** Fragment ions obtained by $MS^2$ and $MS^3$ of *m/z* 758
5. **Figure S2** Characterization of the acid glycosphingolipids (Case 3)
6. **Figure S3** Glycan array, evaluation of serum Anti-S-$Lc_4$ antibodies

**Table S1 - Monoclonal antibodies**

| Antibody | Clone/Designation | Determinant | Manufacturer/Reference | Dilution | Isotype |
|---|---|---|---|---|---|
| Anti-sialyl-lactotetra | TR4 | Neu5Acα3Galβ3GlcNAc | (Svennerholm *et al*., 1989) | 1:1000 | IgM |
| Anti-sialyl-Lewis[a] | 116-NS-19-9 | Neu5Acα3Galβ3(Fucα4)GlcNAc | Signet | 1:30 | IgG1 |
| Anti-NeuAcα3-neolactotetra | LM1:1a | Neu5Acα3Galβ4GlcNAc | (Svennerholm *et al*., 1991) | 1:1000 | IgM |
| Anti-NeuAcα6-neolactotetra | LM4:2 | Neu5Acα6Galβ4GlcNAc | (Nilsson *et al*., 1985) | 1:100 | IgG |

List of antibodies used in the current study, presented with antibody characteristic, reference and applied dilution.

**References**

Svennerholm, L., Boström, K., Fredman, P., Månsson, J.-E., Rosengren, B., and Rynmark, B.-M. (1989) Human brain gangliosides: developmental changes from early fetal stage to advanced age. *Biochim. Biophys. Acta* **1005**, 109-117

Svennerholm, L., Rynmark, B.M., Vilbersson, G., Fredman, P., Gottfries, J., Månsson, J.-E., and Percy, A. (1991) Gangliosides in human fetal brain. *J. Neurochem*. **56**, 1763-1768

Nilsson, O., Lindholm, L., Holmgren, J. and Svennerholm, L. (1985) Monoclonal antibodies raised against NeuAcα2-6neolactotetraosylceramide detect carcinoma-associated gangliosides. *Biochim. Biophys. Acta* **835**, 577-583

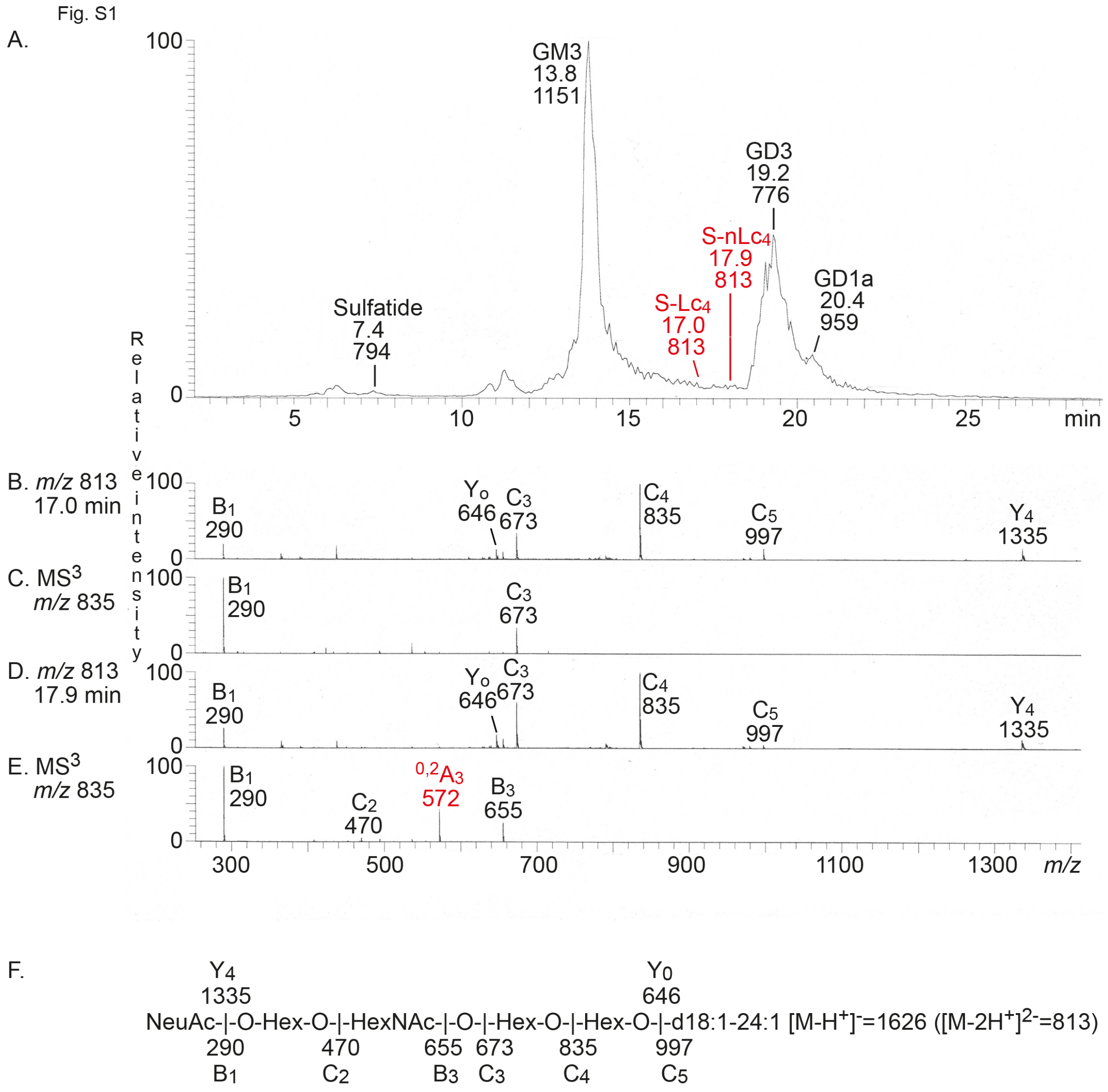


**Figure S1**. **LC-ESI/MS of the total acid glycosphingolipids of ovarian cancer (case 2).** (A) Base peak chromatogram from LC-ESI/MS of acid glycosphingolipids of ovarian cancer (Case 2). (B) $MS^2$ of the ion at *m/z* 813 (retention time 17.0 min) gave series of B and C-type ions at *m/z* 290 ($B_1$), *m/z* 673 ($C_3$), *m/z* 835 ($C_4$), and *m/z* 997 ($C_5$), demonstrating a Neu5Ac-Hex-HexNAc-Hex-Hex sequence. This was corroborated by the Y ions at *m/z* 646 ($Y_o$), and *m/z* 1335 ($Y_4$), also demonstrating a d18:1-24:1 ceramide. (C) $MS^3$ of the ion at *m/z* 835 in (B) gave no $^{0,2}A_3$ ion at *m/z* 572, which indicated a 3-substituted HexNAc, *i.e.* a type 1 carbohydrate chain (Galβ3GlcNAc). Taken together the spectral features in (B) and (C) identified sialyl-lactotetraosylceramide (Neu5Acα3Galβ3GlcNAcβ3Galβ4Glcβ1Cer).
(D) $MS^2$ of the ion at *m/z* 813 (retention time 17.9 min). A Neu5Ac-Hex-HexNAc-Hex-Hex sequence was demonstrated by the series of B and C-type ions at *m/z* 290 ($B_1$), *m/z* 673 ($C_3$), *m/z* 835 ($C_4$), and *m/z* 997 ($C_5$), while the Y ions at *m/z* 646 ($Y_o$), and *m/z* 1335 ($Y_4$) demonstrated a d18:1-24:1 ceramide. (E) $MS^3$ of the ion at *m/z* 835 in (D) gave a $^{0,2}A_3$ ion at *m/z* 572 demonstrating a 4-substituted HexNAc, *i.e.* a type 2 carbohydrate chain (Galβ4GlcNAc). Taken together the spectral features in (D) and (E) identified sialyl-

neolactotetraosylceramide (Neu5Acα3Galβ4GlcNAcβ3Galβ4Glcβ1Cer. (F) Interpretation formula.

The identification of glycosphingolipids was based on their retention times, determined molecular masses and subsequent $MS^2$.

Sulfatide, $SO_3$-3Glcβ1Cer

GM3, Neu5Acα3Galβ4Glcβ1Cer

S-$Lc_4$, Neu5Acα3Galβ3GlcNAcβ3Galβ4Glcβ1Cer (d18:1-16:0 ceramide)

GD3, Neu5Acα8Neu5Acα3Galβ4Glcβ1Cer

S-$nLc_4$, Neu5Acα3Galβ4GlcNAcβ3Galβ4Glcβ1Cer

GD1a, Neu5Acα3Galβ3GalNAcβ4(Neu5Acα3)Galβ4Glcβ1Cer

**Table S2. Theoretical masses and experimental *m/z* of glycosphingolipids isolated from ovarian cancer (Case 1).**

| Glycosphingolipid | Structure | Ceramide | Retention time | $[M+H]^+$ | $[M+2H]^{2+}$ | Observed (*m/z*) |
|---|---|---|---|---|---|---|
| Sulfatide | $SO_3$-3GalCer | d18:1-h16:0 | 12.4 min | 794.51 | - | 794.54 |
| GM3 | Neu5Acα3Galβ4GlcCer | d18:1-16:0 | 16.8 min | 1151.70 | - | 1151.67 |
| S-$Lc_4$ | Neu5Acα3Galβ3GlcNAcβ3Galβ4GlcCer | d18:1-16:0 | 19.5 min | 1516.84 | 758.42 | 758.29 |
| GD3 | Neu5Acα8Neu5Acα3Galβ4GlcCer | d18:1-h24:0 | 21.3 min | 1552.92 | 776.46 | 776.00 |
| S-$nLc_4$ | Neu5Acα3Galβ4GlcNAcβ3Galβ4GlcCer | d18:1-16:0 | 21.8 min | 1516.84 | 758.42 | 758.29 |
| GD1a | Neu5Acα3Galβ4GalNAcβ4(Neu5Acα3)Galβ4GlcCer | d18:1-h24:0 | 22.4 min | 1918.06 | 959.03 | 959.50 |
| S-$nLc_6$ | Neu5Acα3Galβ4GlcNAcβ3Galβ4GlcNAcβ3Galβ4GlcCer | d18:1-16:0 | 24.0 min | 1881.97 | 940.98 | 940.58 |

**Table S3. Fragment ions obtained by $MS^2$ and $MS^3$ of *m/z* 758.**

| **Ion (*m/z*)** | **Proposed ion type** | **Structure** | **Comment** |
|---|---|---|---|
| 290 | $B_1$ | Neu5Ac- | |
| 536 | $Y_0$ | d18:1-16:0-O- | |
| 572 | $^{0,2}A_3$ | Neu5Ac-O-Hex-O-4HexNAc-O- minus 101 | Diagnostic cross-ring $^{0,2}$A-type fragment ion demonstrating a 4-substituted HexNAc |
| 655 | $B_3$ | Neu5Ac-O-Hex-O-HexNAc- | |
| 673 | $C_3$ | Neu5Ac-O-Hex-O-HexNAc-O- | |
| 698 | $Y_1$ | d18:1-16:0-O-Hex-O- | |
| 835 | $C_4$ | Neu5Ac-O-Hex-O-HexNAc-O-Hex-O- | |
| 860 | $Y_2$ | d18:1-16:0-O-Hex-O-Hex-O- | |
| 997 | $C_5$ | Neu5Ac-O-Hex-O-HexNAc-O-Hex-O-Hex-O- | |
| 1225 | $Y_4$ | d18:1-16:0-O-Hex-O-Hex-O-HexNAc-O-Hex-O- | |

Fig. SII

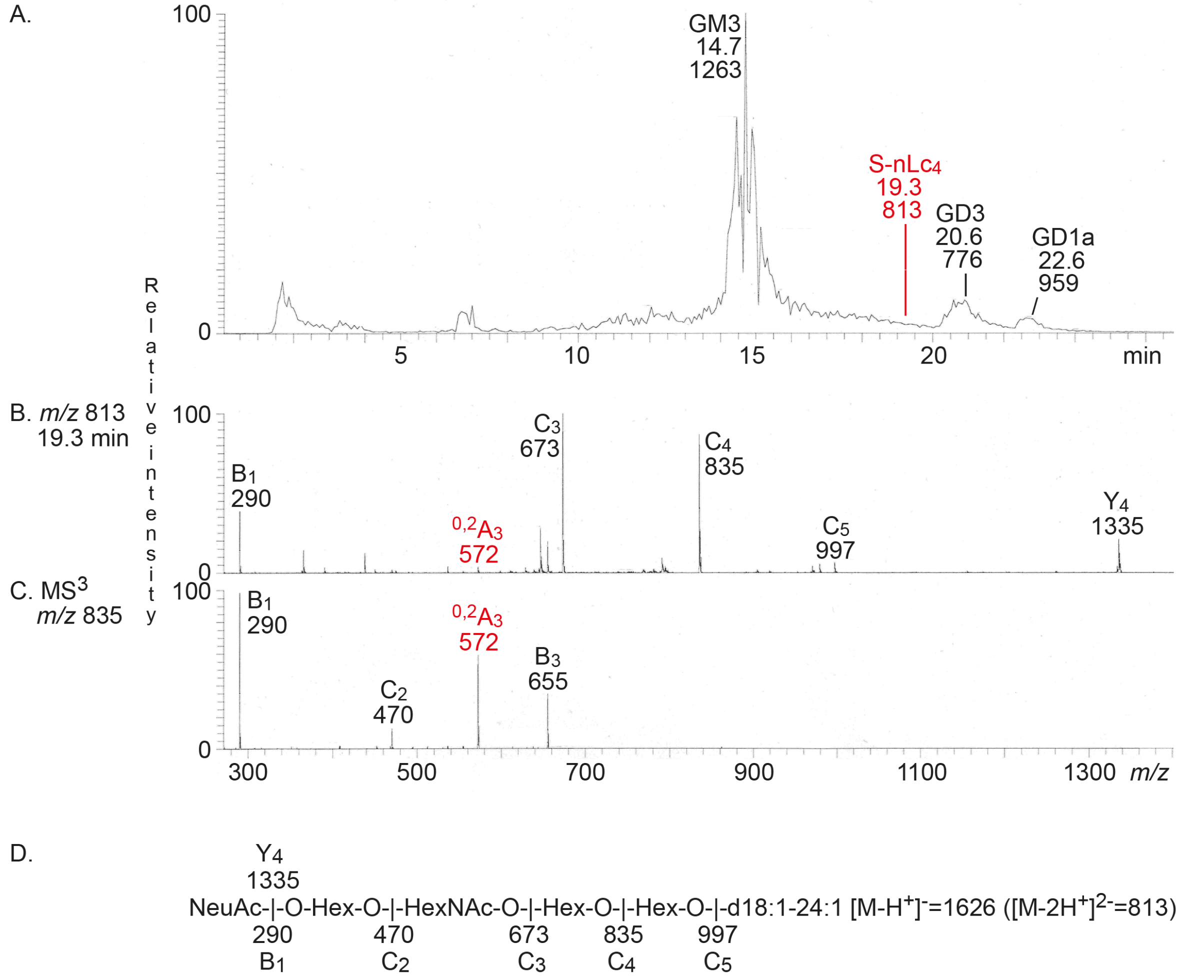


**Figure S2. LC-ESI/MS of the total acid glycosphingolipids of ovarian cancer (Case 3).** (A) Base peak chromatogram from LC-ESI/MS of acid glycosphingolipids of ovarian cancer (Case 3). (B) MS$^2$ of the ion at *m/z* 813 (retention time 19.3 min) gave series of B and C-type ions at *m/z* 290 ($B_1$), *m/z* 673 ($C_3$), *m/z* 835 ($C_4$), and *m/z* 997 ($C_5$), demonstrating a Neu5Ac-Hex-HexNAc-Hex-Hex sequence. This was corroborated by the Y ion at *m/z* 1335 ($Y_4$), also demonstrating a d18:1-24:1 ceramide. (C) MS$^3$ of the ion at *m/z* 835 in (B) gave a $^{0,2}A_3$ ion at *m/z* 572 demonstrating a 4-substituted HexNAc, *i.e.* a type 2 carbohydrate chain (Galβ4GlcNAc). Taken together the spectral features in (B) and (C) identified sialyl-neolactotetraosylceramide (Neu5Acα3Galβ4GlcNAcβ3Galβ4Glcβ1Cer). (D) Interpretation formula.

The identification of glycosphingolipids was based on their retention times, determined molecular masses and subsequent MS$^2$.

GM3, Neu5Acα3Galβ4Glcβ1Cer (d18:1-24:0 ceramide)

GD3, Neu5Acα8Neu5Acα3Galβ4Glcβ1Cer

S-nLc$_4$, Neu5Acα3Galβ4GlcNAcβ3Galβ4Glcβ1Cer

GD1a, Neu5Acα3Galβ3GalNAcβ4(Neu5Acα3)Galβ4Glcβ1Cer

**A**

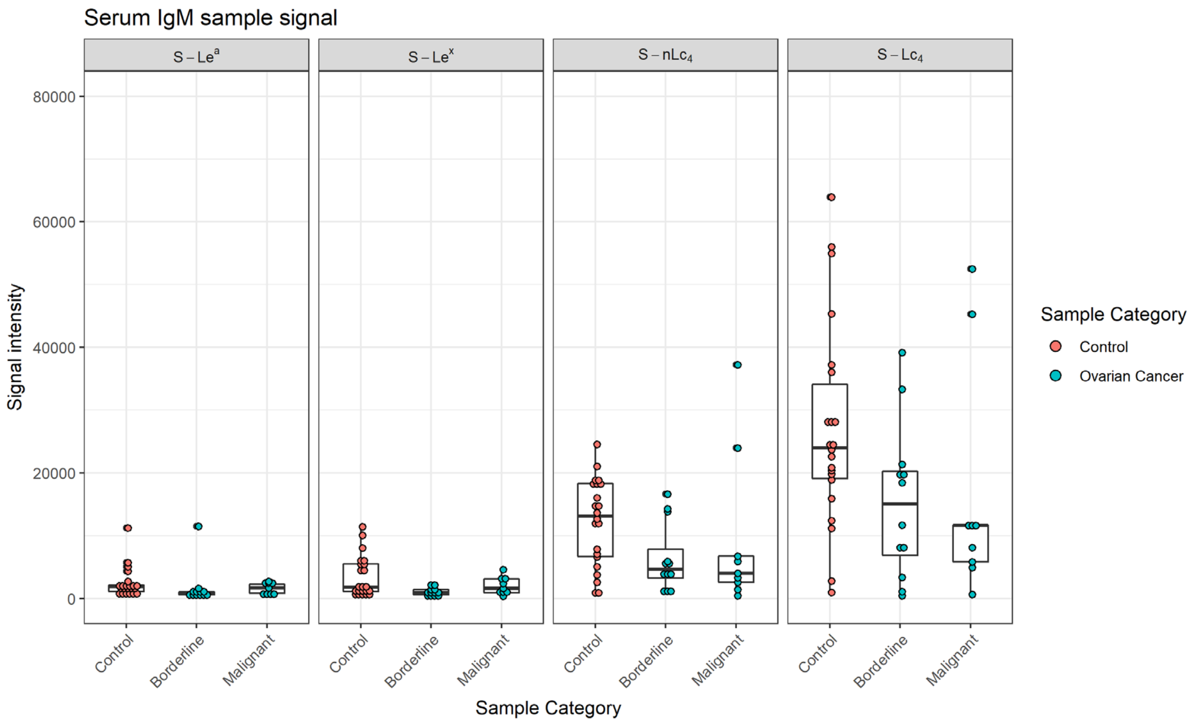


**B**

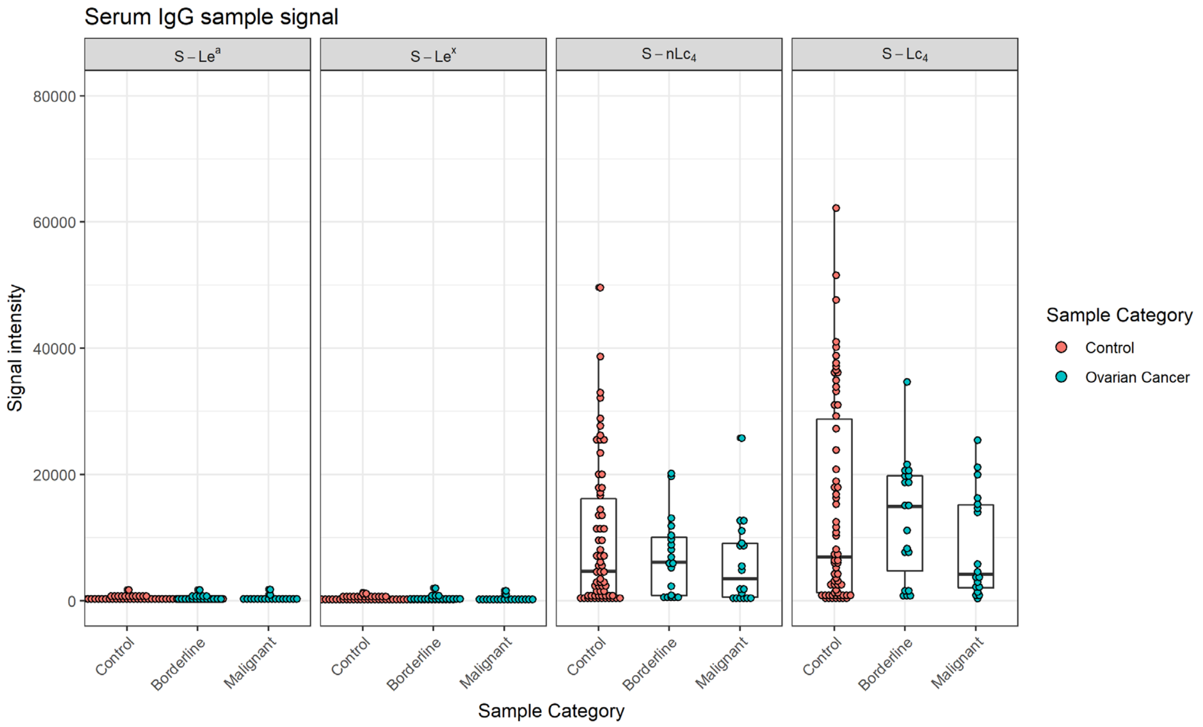


**Figure S3** Glycan array
(A) Box/dot plots displaying the IgG reactivity of control and ovarian cancer serum samples towards four carbohydrate structures. S-Le$^a$ and S-Le$^x$ gave no IgG reactivity. S-Lc$_4$ and S-nLc$_4$ gave some reactivity but there was no significant difference compared with the control. (B) Same as (A), but displaying IgM reactivity. Again, -Le$^a$ and S-Le$^x$ gave no IgM reactivity S-Lc$_4$ and S-nLc$_4$ gave increased reactivity, but the level of controls seemed to be higher than that of ovarian tumour samples. However, it should be noted that the sample size was smaller in the IgM experiment.

Samples included in the analysis, IgG; Control *n* = 66, borderline tumours *n* = 19 (9 negative + 10 positive), malignant tumours *n* = 18 (10 negative + 8 positive). IgM; Control *n* = 22, borderline tumours *n* = 12 (6 negative + 6 positive) and malignant tumours *n* = 9 (4 negative + 5 positive).

Groups were compared using the non-parametric Mann-Whitney U test.

SiaLea, Neu5Aca2-3Galb1-3(Fuca1-4)GlcNAcb-sp3

SiaLex, Neu5Aca2-3Galb1-4(Fuca1-3)GlcNAcb-sp3

S-nLc$_4$, Neu5Aa2-3Galb1-4GlcNAcb1-3Galb1-4Glcb-sp4

S-Lc$_4$, Neu5Aa2-3Galb1-3GlcNAcb1-3Galb1-4Glcb-sp4

(sp3 and sp4 being functional groups that allow for covalent binding on microarray surface)
